# Telomerase as a therapeutic Bullseye: advances in cancer treatment and clinical trials: A comprehensive review of small molecule inhibitors, antisense oligonucleotides, and immunotherapeutic strategies targeting the telomerase catalytic subunit (hTERT)

**DOI:** 10.1186/s40659-026-00715-9

**Published:** 2026-07-03

**Authors:** Ansar Hussain, Musavir Abbas, Ming Long, Ghulam Mustafa, Hafiz Muhammad Yasir, Tanveer Abbas, Yousaf Raza, Muhammad Lateef, Muhammad Shoaib, Zain Ul Abideen, Wasim Shah

**Affiliations:** 1 Chongqing Precision Medical Industry Technology Research Institute, Chongqing, 400000 China; 2 Centre for Reproduction and Genetics, First Affiliated Hospital of USTC, Hefei National Laboratory for Physical Sciences at Microscale, School of Basic Medical Sciences, Biomedical Sciences and Health Laboratory of Anhui Province, Institute of Health and Medicine, Hefei Comprehensive National Science Centre, Division of Life Sciences and Medicine, University of Science and Technology of China, Hefei, China

**Keywords:** Telomerase inhibition, Cancer treatment, Small molecule inhibitors, Immunotherapy, Targeted cancer treatment

## Abstract

Telomeres play a crucial role in maintaining genomic stability in healthy cells. However, they gradually shorten during the cell cycle, leading to chromosomal instability. Telomere length and telomerase activity are vital factors that counteract cellular degradation in cancer development and tumor persistence. Telomerase, which is activated in most cancer cells due to telomerase catalytic subunit (hTERT) overexpression, serves as a universal biomarker that is essential for cancer cell growth and survival. The upregulation of hTERT, often associated with G > A mutations in its promoter region, is frequently implicated in cancer progression. Consequently, anti-telomerase therapy has been proposed as a potentially more efficacious alternative to conventional treatment. Small-molecule inhibitors have garnered significant attention owing to their selectivity or ability to modulate multiple proteins. However, challenges, such as low response rates, brief response durations, toxicity, and resistance persist. Several strategies have been proposed to target telomerase activity and the telomere structure. These include the utilization of G-quadruplex-stabilizing compounds and telomere-specific oligonucleotide inhibitors of telomerase such as GRN163L and T-oligos. Another therapeutic approach involves the use of biological antisense oligonucleotides that specifically inhibit hTERT and human telomerase RNA component genes, potentially reducing telomerase activity and generating robust DNA signals in cancer cells. Immunotherapy targeting hTERT represents a recent advancement in cancer treatments. This approach leverages the immune system to target cancer cells with high hTERT expression, thereby offering a potentially more reliable treatment strategy. This review provides an overview of current research on telomerase-targeting small-molecule inhibitors, antisense oligonucleotides, and immunotherapy, discussing their mechanisms, clinical applications, and prospects in cancer treatment.

## Background

**Fig. 1:**
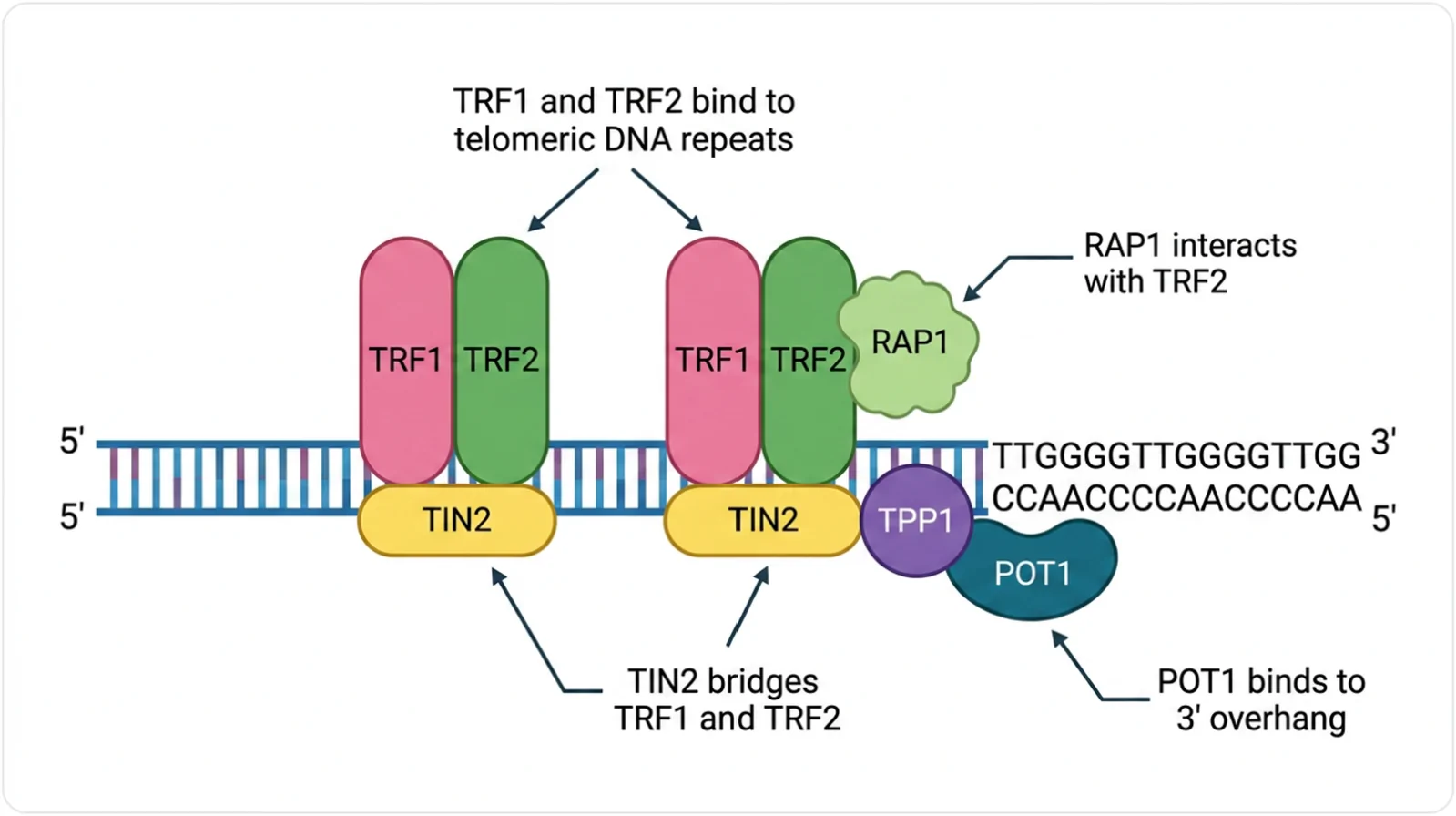
Schematic representation of the shelterin complex architecture at mammalian telomeres

The integrity of the human genome depends critically on specialized nucleoprotein structures that cap the termini of linear chromosomes-the telomeres. In humans, telomeric DNA consists of tandem repeats of the hexanucleotide sequence 5′‑TTAGGG‑3′, typically spanning 10-15 kb, although recent pan‑cancer genomic analyses have revealed variant repeats such as 5′‑TGAGGG‑3′, suggesting greater sequence heterogeneity than previously recognized. These repetitive tracts are bound by the shelterin complex, a six‑protein assembly (TRF1, TRF2, POT1, TIN2, TPP1, and RAP1) that distinguishes telomeric DNA from sites of double‑strand breaks and actively suppresses inappropriate DNA damage responses, thereby preserving chromosomal stability during successive cell divisions (Fig. 1) [1–4].

This schematic illustrates the hierarchical organization of the shelterin complex bound to telomeric DNA. The core DNA platform consists of double-stranded telomeric repeats (depicted with the sequences 5’-TGGGGTTGGGGTTGG-3’ and its complementary strand 3’-CCAACCCCAACCCCA-5’). TRF1 and TRF2 directly bind to these double-stranded telomeric tracts as homodimers. RAP1 is recruited to the complex specifically via its protein–protein interaction with TRF2 but does not contact the DNA itself. At the distal end, the single-stranded 3’ G-rich overhang is bound by POT1, which functions to protect the chromosome terminus from inappropriate DNA damage responses. The complex is stabilized through TIN2, which acts as a critical bridging factor by simultaneously interacting with both TRF1 and TRF2, thereby tethering the components together and maintaining the structural integrity of the telomere cap.

A fundamental constraint of cellular replication is the progressive attrition of telomeres that occurs with each mitotic cycle, a consequence of the end‑replication problem. When telomeres become critically short, cells enter replicative senescence-a potent tumor‑suppressive barrier that limits the proliferative potential of somatic tissues. This barrier is overcome in highly replicative cells by the ribonucleoprotein enzyme telomerase, which counteracts telomere erosion by processively adding TTAGGG repeats to chromosome ends [5]. The human telomerase holoenzyme comprises a catalytic protein subunit-telomerase reverse transcriptase (TERT)-an RNA template component (TR), and associated accessory factors including WRAP53 and the H/ACA ribonucleoprotein complex (dyskerin, NOP10, NHP2, and Gar1) [5–11]. While TERT expression is tightly restricted to embryonic, germline, and adult stem cells, TERC is transcribed ubiquitously; thus, telomerase activity is tightly regulated at the level of TERT availability [11, 12]. Importantly, TERT exerts a context‑dependent dual function it can either promote or suppress tumorigenesis depending on telomere length and the cellular milieu underscoring its role as a gatekeeper of replicative lifespan [13].

To achieve unlimited proliferation-a hallmark of malignancy-cancer cells must activate telomere maintenance mechanisms (TMMs) that circumvent replicative senescence [14, 15]. Approximately 85% of human cancers accomplish this by reactivating telomerase, thereby restoring telomere elongation and enabling continuous division [15]. The remaining tumors, including many sarcomas and a subset of carcinomas, employ an alternative lengthening of telomeres (ALT) pathway that relies on homologous recombination between telomeric sequences [16, 17]. These two TMMs are spatially and mechanistically distinct: telomerase‑dependent maintenance requires Cajal bodies for holoenzyme maturation and telomere recruitment, whereas ALT is orchestrated within ALT‑associated PML bodies (APBs) and is driven by replication‑stress‑induced recombination [16, 18–25]. Notably, TMMs are not mutually exclusive and can coexist in a mosaic pattern both across tumors and within individual cancer cells, a complexity that has important implications for therapeutic strategies [26–29]. Droplet-based single-cell RNA sequencing (scRNA-seq) has recently emerged as a powerful approach to resolve such inter- and intra-tumoral heterogeneity, offering unprecedented resolution to dissect the coexistence of telomerase-dependent and ALT-driven populations within individual tumors [30]. Moreover, genetic or pharmacological inhibition of telomerase can drive cancer cells to switch to the ALT pathway, highlighting the inherent plasticity of telomere maintenance and the need for combinatorial approaches [31–34].

The modulation of telomerase catalytic subunit (hTERT) activity has been predominantly elucidated through holoenzyme assembly, cellular compartmentalization, and transcriptional regulation. Concurrently, it has been established that point mutations in the hTERT promoter region are detected in a substantial proportion of patients with various forms of cancer, including approximately 83% of glioblastoma, 70% of hepatocellular carcinoma, 85% of bladder cancer, up to 75% of skin tumors, including melanoma and non-melanoma, and 80% of urinary tract tumors. In all of these instances, these mutations were G > A transitions and were observed to enhance telomerase hyperactivation, poor prognosis, and tumor recurrence [35].

Given its near‑universal expression in malignancies and its essential role in sustaining cancer cell proliferation, telomerase represents an exceptionally attractive therapeutic target. However, conventional treatments often suffer from limited response rates, acquired resistance, and off‑target toxicity. In response, a diverse arsenal of telomerase‑directed strategies has been developed, including small‑molecule kinase inhibitors, antisense oligonucleotides (such as the recent FDA‑approved imetelstat for lower‑risk myelodysplastic syndromes), G‑quadruplex‑stabilizing ligands, and immunotherapeutic approaches that leverage the immune system to eliminate hTERT‑expressing tumor cells. This review provides a comprehensive synthesis of current research on these modalities, critically discussing their mechanisms of action, clinical trial outcomes, and the challenges including drug resistance, alternative TMM activation, and safety concerns that must be addressed to translate telomerase‑targeted therapies into durable clinical benefit for cancer patients.

## Telomerase inhibition strategies

Telomerase inhibition has emerged as a promising strategy in cancer therapy, particularly due to the enzyme’s critical role in maintaining telomere length and enabling the unlimited proliferation of cancer cells. Recent advancements in understanding telomerase biology have led to the development of novel approaches for early cancer detection like diagnosing liver cancer through amplification of mutational extracellular mRNA [36] and targeted treatment. Various strategies, such as small molecule inhibitors, antisense oligonucleotides, and immunotherapeutic agents, have been explored to disrupt telomerase activity. Despite significant progress, challenges remain, including cancer cell resistance mechanisms and the need for selective targeting to minimize off-target effects on normal cells. Understanding these complexities is essential for designing effective telomerase inhibition therapies and improving patient outcomes in cancer treatment.

### Small molecule inhibitors

#### Protein kinase inhibitors

**Fig. 2:**
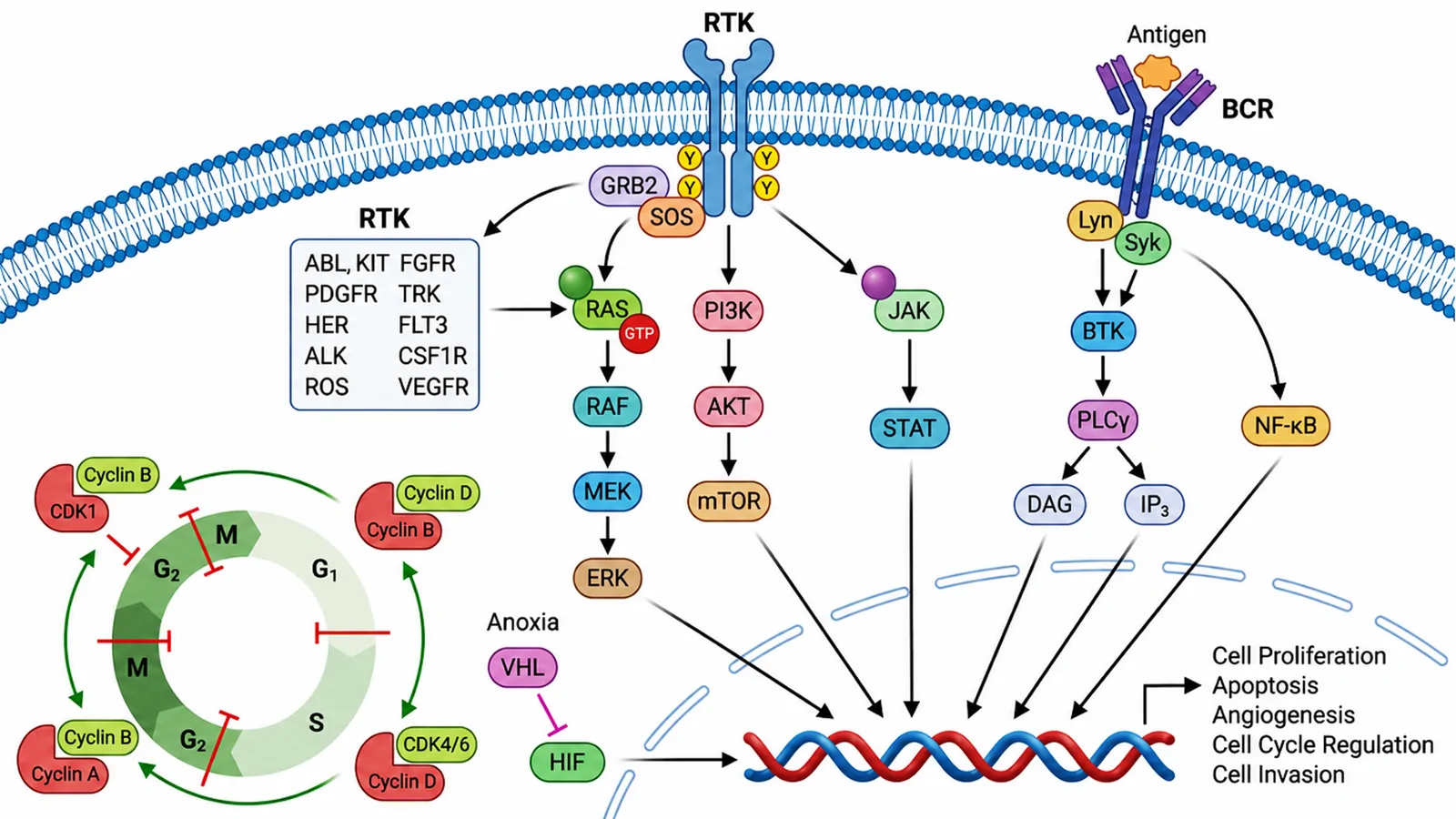
Schematic representation of signaling convergence on CDK regulation

Protein kinases are enzymes that transfer the γ-phosphate of ATP to hydroxyl-containing amino acids in proteins, playing a crucial role in regulating cell division, differentiation, and growth. Dysregulated kinase activity is a hallmark of cancer, as it leads to uncontrolled cell proliferation, survival, and metastasis, making these enzymes critical therapeutic targets. The human kinome comprises approximately 535 protein kinases, categorized based on their target substrates, including receptor/non-receptor tyrosine kinases, serine/threonine kinases, and TKL enzymes. Aberrant kinase signaling arises through multiple mechanisms in cancer, including activating mutations, gene amplifications, chromosomal translocations, and autocrine/paracrine growth factor loops. Protein kinase inhibitors are classified into six types (I-VI) based on their binding mechanisms: Type I inhibitors target the active kinase conformation (inward DFG-Asp, αC-helix), while Type I½ inhibitors target the inactive conformation (inward DFG-Asp, outward αC-helix), both interacting with the adenine-binding pocket and hinge region. Type II inhibitors are subdivided into classes A (penetrating the back cleft with longer residence time) and B. Type III and IV inhibitors are allosteric, with Type III binding near the ATP site and Type IV binding outside the cleft. Type V inhibitors are bidentate, interacting at two distinct sites within the kinase domain, and Type VI inhibitors form permanent covalent bonds at the active site. These inhibitors are critical in cancer treatment by targeting aberrant kinase activity (Fig. 2).

Extracellular ligands and antigens engage RTKs (e.g., ABL, KIT, HER, FGFR) and immunoreceptors (via IgY), triggering downstream phosphorylation cascades involving Lyn/Syk/BTK, PI3K-PI3P, PLCγ-IP₃/DAG, and IKK, alongside stress-responsive p38/JNK MAPKs. These pathways integrate to ultimately control Cyclin-dependent kinases (highlighting CDK1, CDK2, CDK4/6, CDK7, and CDK9) that drive cell cycle progression.

### Inhibitors targeting receptor tyrosine kinase

#### Anaplastic lymphoma kinase inhibitor

Anaplastic Lymphoma Kinase (ALK), a member of the insulin receptor-related kinase family, is encoded by a single transmembrane tyrosine kinase gene and plays a crucial role in neurodevelopment by regulating various signaling pathways [6, 37]. ALK rearrangements have been identified in several cancers, including anaplastic large-cell lymphoma, DLBCL, inflammatory myofibroblastic tumors, and NSCLC [38–40]. In NSCLC, EML4-ALK fusion occurs through chromosomal inversion, resulting in ALK activation and cellular proliferation in 3–7% of cases [40].

The first-generation ALK inhibitor crizotinib, approved in 2011, demonstrated enhanced efficacy in ALK-positive NSCLC patients. However, resistance mutations typically emerge within a year, predominantly in the central nervous system, owing to poor blood-brain barrier (BBB) penetration. Among second-generation inhibitors, ceritinib is the most potent, but alectinib, which is capable of crossing the BBB and targeting multiple resistant ALK mutations, is recommended as a first-line therapy [41]. Brigatinib, approved in 2017, exhibits high efficacy in both systemic and intracranial settings [42]. Lorlatinib, a third-generation inhibitor introduced in 2018, is effective against all known ALK mutants, except L1198F, which can be re-sensitized to crizotinib [43–45]. Additional ALK inhibitors under investigation include entrectinib, belizatinib, repotrectinib, and ensartinib with ongoing clinical trials [46–48]. Ensartinib, designed for both treatment-naïve and crizotinib-resistant patients, is currently undergoing a phase III trial comparing it to crizotinib [48]. Drug-resistant mutations present a significant challenge for ALK inhibitor treatment. Resistance can be addressed through sequential application of first-, second-, and third-generation inhibitors. Alternative strategies encompass the combination of ALK inhibitors with other targeted molecular therapies and the utilization of PROTAC technology for ALK protein elimination [49–52].

#### The potential of c-Met inhibitors

Located on chromosome 7q21-31, the MET proto-oncogene codes for the cytokine receptor involved in mesenchymal to epithelial cell conversion. When HGF binds to c-Met, it triggers various signaling cascades, including PI3K/AKT, MAPK, STATs, and NF-κB. These activated pathways contribute to multiple cellular processes, such as proliferation, survival, invasion, motility, angiogenesis, and the transformation of epithelial cells into mesenchymal cells [53]. While these processes are physiological during embryonic development and tissue remodeling, c-MET dysregulation in neoplasms, primarily due to increased copy numbers through MET gene amplification, point mutations, or other unidentified mechanisms in various solid malignancies, has been associated with poor outcomes in the context of resistance to EGFR inhibitors [54].

Selective c-Met inhibitors, including capmatinib and tepotinib, were introduced at the end of 2019 and the beginning of 2020, with both receiving FDA approval. Notably, Capmatinib demonstrated significant c-Met selectivity and yielded promising response rates in a mono-1 trial, which involved mNSCLC patients with MET exon 14-skipping mutations or brain metastasis [55]. A phase II trial combining capmatinib and gefitinib achieved an overall disease control rate of 80%. Tepotinib, another therapeutic agent, exhibited efficacy in a phase II trial and was subsequently approved in Japan for the treatment of MET exon 14 skipping mutations in NSCLC.

Multiple small-molecule compounds targeting c-Met are currently undergoing clinical trials. These include multi-target kinase inhibitors such as foretinib (XL880/GSK1363089), glesatinib (MGCD265), BMS-777,607, and S49076, which additionally target RON, VEGFR-2, KIT, TIE2, PDGFR, AXL, and MER. Although these inhibitors have been utilized in numerous clinical trials for cancer therapy, comprehensive reports are yet to be published. For instance, a phase I trial combining foretinib with erlotinib in previously chemotherapy-treated NSCLC patients with NSCLC (NCT01068587) demonstrated a 17% objective response rate. Zhu and colleagues (2019) reported an 8% response rate correlated with baseline c-Met expression. Similarly, S49076 exhibited moderate to minimal adverse effects and a 23% overall response rate in a phase I study of advanced solid malignancies (ISRCTN00759419) [56].

Several other c-Met-specific inhibitors are currently at various stages of development. Tivantinib demonstrated promising outcomes in a phase II study involving previously treated patients with NSCLC (NCT00777309) [26, 57]. Researchers have investigated volitinib (savolitinib) in combination with gefitinib or osimertinib for NSCLC, yielding positive initial results [58]. SAR125844, a triazolopyridazine c-MET kinase inhibitor, exhibited acceptable activity against NSCLC in phase I trials with patients demonstrating MET amplification (NCT01657214) [59]. Additionally, multiple c-Met inhibitors, including bozitinib, ningetinib, glumetinib, and kanitinib, are currently undergoing clinical trials in China, and may potentially receive approval in the future.

In addition to small molecules, antibodies targeting HGF ligands and c-Met receptors serve as another efficacious approach to counteract the HGF/c-Met effects [60]. Currently, the primary challenge lies in identifying which patients respond optimally to c-Met-targeted therapies. Factors such as MET mutations, gene copy number, and HGF protein expression are crucial for determining the efficacy of c-Met inhibitors in patient samples [61]. For instance, the presence of the MET exon 14 skipping mutation aids in predicting the effectiveness of treatments such as capmatinib or tepotinib [62]. Challenges in c-Met inhibitor treatment include intrinsic and acquired drug resistance mechanisms, MET or KRAS amplification, and secondary mutations [63]. Nevertheless, novel combinations of HGF-neutralizing antibodies and other targeted therapies may prove more efficacious and assist in overcoming resistance in various patient populations [64].

#### EGFR inhibitors

The epidermal growth factor receptor (EGFR) is a crucial transmembrane protein that regulates various signal transduction pathways. This receptor family, which includes ERBB2/HER2, ERBB3/HER3, and ERBB4/HER4, comprises an extramembrane ligand-binding domain, single transmembrane helix, and intracellular tyrosine kinase domain [65]. When ligands, such as EGF and TGF-α, bind to EGFR, they initiate receptor dimerization and autophosphorylation of the tyrosine kinase domain, activating downstream signaling pathways essential for growth, survival, and apoptosis [66]. EGFR genes are associated with the progression of several cancer types, including lung, breast, and pancreatic [67].

Various EGFR-targeting tyrosine kinase inhibitors (TKIs) have been developed and are currently available. First-generation EGFR TKIs, including gefitinib, erlotinib, and icotinib, are reversible and exhibit the highest efficacy in non-small cell lung cancer (NSCLC) patients with EGFR-activating mutations such as exon 19 deletions and exon 21 L858R mutations [68, 69]. However, the emergence of the L858R/T790M mutation frequently leads to resistance in many treated cases, thereby diminishing its effectiveness.

Afatinib and dacomitinib, classified as second-generation irreversible EGFR-TKIs, are designed to target the T790M mutation. These medications establish covalent connections with EGFR’s ATP-binding site of EGFR, resulting in a pharmacological profile that demonstrates greater efficacy than first-generation alternatives. However, a key limitation of these second-generation inhibitors is their significant off-target activity against wild-type EGFR, which leads to severe adverse effects, including skin rash and diarrhea, often necessitating dose reductions and limiting their overall therapeutic window. Furthermore, their irreversible binding mechanism, while providing sustained target inhibition, also contributes to the severity and persistence of these side effects. This toxicity profile has restricted their clinical utility, particularly in patients with pre-existing skin conditions or gastrointestinal disorders and has prompted the development of more selective third-generation inhibitors. The emergence of the L858R/T790M mutation frequently leads to resistance in many treated cases [70–72]. Osimertinib, a third-generation EGFR TKI, specifically inhibits EGFR-activating mutations and T790M, demonstrating superior efficacy for NSCLC patients with NSCLC resistant to first- and second-generation EGFR-TKIs [73]. Almonertinib, another drug in the same molecular class as osimertinib, has demonstrated potential in clinical trials for metastatic NSCLC that has developed resistance to first-line therapy [74]. These third-generation inhibitors utilize their enhanced selectivity and affinity for the T790M mutation, effectively overcoming the acquired resistance mechanisms.

Clinical approval has been granted for dual inhibitors that simultaneously target EGFR and HER2 in breast cancer treatment, with lapatinib and neratinib being notable examples [75, 76]. The use of lapatinib in combination with capecitabine has been proven effective in managing advanced or metastatic HER2-positive breast cancer cases. Conversely, neratinib is primarily indicated for patients who do not benefit from other HER2-targeted treatment options [77]. Several novel EGFR inhibitors are currently being investigated. Olmutinib, for instance, exhibits potent activity against the L858R/T790M mutation but has limited utility due to significant adverse effects [78]. Avitinib and pelitinib are also undergoing evaluation in clinical trials for their efficacy against EGFR mutations in NSCLC and other malignancies [79, 80].

The development of resistance to EGFR-TKIs remains a significant challenge, typically manifesting within 9–14 months after treatment initiation. This resistance can arise from various sources, including secondary mutations in EGFR and activation of alternative signaling pathways [81]. Current scientific investigations are focused on the development of fourth-generation EGFR TKIs and innovative inhibitors designed to overcome these resistance mechanisms [82].

#### FLT3 tyrosine kinase inhibitors

FLT3, also known as FLK2 or STK-1, is a membrane-spanning receptor tyrosine kinase encoded by the FLT3 proto-oncogene. It is a member of the type III receptor tyrosine kinase family, which also includes PDGFR, FMS, and KIT [83]. Upon ligand binding, FLT3 dimerizes and undergoes autophosphorylation of its tyrosine residues. This process activates downstream signaling pathways, including PI3K/AKT/mTOR, RAS/RAF/MAPK, and JAK/STAT, which are responsible for promoting cell growth, differentiation, and survival [84]. In AML patients, FLT3 is expressed at elevated levels, with 25% of cases exhibiting ITD mutations and 7-10% displaying TKD mutations. These mutations play critical roles in leukemogenesis.

To address this issue, researchers have developed various inhibitors targeting FLT3 mutations. The first generation of inhibitors, including sorafenib and sunitinib, targeted multiple kinases, but demonstrated limited efficacy due to adverse effects. In 2017, the FDA approved midostaurin, a first-generation inhibitor, for use in patients with FLT3-mutated AML. This drug has demonstrated improved overall survival rates when administered in conjunction with conventional chemotherapy treatments. Pexidartinib, a multi-targeted TKI approved for GCTs, interacts with FLT3, but is not utilized in AML treatment. Second-generation FLT3 inhibitors such as gilteritinib and quizartinib have been designed to increase specificity and potency. Gilteritinib demonstrated significantly longer median overall survival compared to conventional chemotherapy in relapsed or refractory AML patients with FLT3 mutations, while quizartinib exhibited activity against FLT3-ITD mutations [85]. Ongoing research is investigating the efficacy of crenolanib in combination treatment approaches, particularly for AML cases that have recurred or become resistant to other therapies [83].

Resistance to FLT3 inhibitors, driven by primary mutations and alternative signaling pathways, remains a significant challenge. Strategies to overcome resistance include the combination of therapies with epigenetic modulators and inhibitors that target downstream pathways. Novel approaches, such as irreversible FLT3 inhibitors such as FF-10,101, are being investigated to address secondary mutations, such as the F691L gatekeeper mutation [85]. Research on FLT3 inhibitors continues, with promising candidates such as SKLB-1028 and various others in preclinical stages, aiming to improve outcomes in AML patients with FLT3 mutations.

#### VEGFR/FGFR/PDGFR inhibitors

Angiogenesis, the critical phase in which new blood vessels form, plays a significant role in the progression of various diseases, including cancer. In cancer, these newly formed blood vessels support and facilitate tumor growth and metastasis. Anti-angiogenic therapy has evolved from suppressing angiogenesis to specifically targeting certain receptor tyrosine kinases (RTKs) involved in angiogenic signaling pathways. Some of the most frequently targeted receptors include VEGFR-1-3, FGFR1-4, PDGFRα, and PDGFRβ [86–90].

The FDA and NMPA have approved various multi-targeted tyrosine kinase inhibitors that target specific receptors, demonstrating efficacy in treating different solid tumors. The first anti-angiogenic therapy approved for advanced RCC, HCC, and DTC was sorafenib, which targets VEGFR-1/2/3, c-Kit, FLT3, RET, and PDGFRβ [91–94]. Another approved drug, sunitinib, which affects VEGFR-1/2/3, PDGFRα/β, c-Kit, CSF1R, RET, and FLT3, is used for RCC and imatinib-resistant GIST. Vandetanib is employed to treat metastatic medullary thyroid cancer (MTC) by inhibiting EGFR, VEGFR-2/3, RET, BRK, TIE2, and EPH [95]. Furthermore, regorafenib, which targets VEGFR-1/2/3, PDGFRα/β, FGFR1/2, BRAF, c-Kit, and RET, has been approved for metastatic colorectal cancer (mCRC) and advanced GIST [96]. Novel selective inhibitors have expanded the therapeutic options, such as erdafitinib for urothelial carcinoma [97], pemigatinib for cholangiocarcinoma [98], and avapritinib for GIST with PDGFRα exon 18 mutations. These agents underscore their selectivity and effectiveness in treating tumors with FGFR or PDGFR dependency. Numerous ongoing clinical trials are investigating other newly developed inhibitors of VEGFR, FGFR, PDGFR, and TGF-β receptors with the objective of establishing improved therapeutic effects and indications [99].

Despite progress, challenges remain in maximizing clinical benefits and identifying biomarkers for patient selection. Combination therapies, such as axitinib plus pembrolizumab for RCC [100], represent promising strategies to enhance efficacy by targeting complementary pathways and overcoming resistance mechanisms. The complexity of angiogenic signaling and its interaction with immune responses necessitate ongoing research into biomarkers and personalized treatment approaches to optimize outcomes.

#### TRK inhibitors

The TRK family comprises of three proteins: TRKA, TRKB, and TRKC. These proteins are encoded by the NTRK1, NTRK2, and NTRK3 genes. The interaction of neurotrophins with these receptors results in receptor dimerization and autophosphorylation, subsequently activating the RAS/MAPK/ERK and PI3K/AKT pathways [101]. NTRK gene rearrangements produce oncogenic fusion proteins, rendering TRKs important in cancer therapy [102]. Although NTRK fusions are present in only 1% of all cancers, they occur frequently in certain types such as infantile fibrosarcoma and secretory breast carcinoma.

Larotrectinib and entrectinib, two first-generation TRK inhibitors, have demonstrated high efficacy in treating TRK fusion cancers and have received FDA approval [103]. These drugs exhibit both safety and efficacy in CNS tumors with NTRK fusions. Second-generation TRK inhibitors targeting multiple kinases are currently being tested to address [104].

Resistance to TRK inhibition primarily occurs through mutations in the kinase domain of the NTRK fusions. To address this challenge, researchers have developed advanced TRK inhibitors including selitrectinib and repotrectinib. These novel compounds are currently being evaluated in phase I/II clinical trials [105].

## Targeting non-receptor tyrosine kinases

### Bcr-Abl1 viral oncogene inhibitors

ABL1, located on chromosome 9q34, encodes c-Abl, a non-receptor tyrosine kinase protein. This protein plays a critical role in regulating cell differentiation, cell cycle progression, and cell viability [106]. The Philadelphia (Ph) chromosome results from a t(9;22)(q34;q11) translocation, which fuses the ABL1 gene with the breakpoint cluster region (BCR) gene on chromosome 22. This fusion leads to the formation of the BCR-ABL fusion gene [107]. The resulting p210 Bcr-Abl1 oncoprotein exhibits constitutive tyrosine kinase activity, promoting the uncontrolled proliferation of leukemic cells. This phenomenon is particularly significant in CML and ALL [108].

The BCR-ABL fusion serves as a crucial diagnostic marker and treatment response indicator in these malignancies, positioning Bcr-Abl1 tyrosine kinase as a primary therapeutic target [109]. Imatinib, a pioneering small-molecule TKI targeting Bcr-Abl1, represents a significant advancement in targeted cancer therapy [110]. It has demonstrated remarkable efficacy in the treatment of CML-CP and Ph + ALL, with long-term studies reporting OS and PFS rates exceeding 89% and 93%, respectively.

The development of resistance poses a significant challenge to imatinib treatment, primarily because of point mutations occurring in the BCR-ABL kinase domain [111]. Several mutations, including G250E, Q252H, Y253H/F, E255K/V, T315I, and H395P/R, impair imatinib’s ability to bind to Bcr-Abl1, thereby reducing its inhibitory efficacy [112]. In response to this resistance, researchers have introduced second-generation Bcr-Abl1 inhibitors, including dasatinib, nilotinib, bosutinib, and radotinib, which were approved between 2006 and 2012. Subsequently, third-generation inhibitors, such as ponatinib, were developed, demonstrating increased affinity for various BCR-ABL mutants, although they remain ineffective against the T315I gatekeeper mutation [113]. Ponatinib, specifically designed to overcome the T315I mutation, has been approved for CML and ALL patients who are resistant or intolerant of other Bcr-Abl1 inhibitors.

Current research focuses on the development of novel Bcr-Abl1 inhibitors to address emerging resistance mechanisms. Asciminib (ABL001), an allosteric Abl1 inhibitor targeting the myristoyl pocket to induce an inactive kinase state, and rebastinib (DCC-2036), a conformational control inhibitor effective against T315I mutations, are currently undergoing clinical evaluation [114].

Despite these advancements, challenges persist, including sequential therapy-induced mutations and compound mutations that affect CML treatment outcomes. Management strategies involve the rotation of available inhibitors based on mutational profiles, with omacetaxine mepesuccinate providing an alternative for patients with T315I mutations who are unable to tolerate ponatinib owing to cardiovascular concerns [115]. Moreover, imatinib, dasatinib, and nilotinib have demonstrated efficacy in the treatment of other malignancies driven by c-Kit, PDGFR, or Src mutations, such as gastrointestinal stromal tumors (GISTs) and systemic mastocytosis [116, 117].

### BTK inhibitors

The B-cell receptor (BCR) pathway is of paramount importance in various B-cell malignancies, including B-cell non-Hodgkin’s lymphoma (NHL), chronic lymphocytic leukemia (CLL), and mantle cell lymphoma [80]. Bruton’s agammaglobulinemia tyrosine kinase (BTK), a crucial element of this pathway and a member of the TEC family of non-receptor tyrosine kinases, governs B-cell proliferation and viability in these cancers [118]. The seminal irreversible BTK inhibitor ibrutinib forms a covalent bond with Cys-481 in the ATP-binding pocket of BTK. Clinical trials have demonstrated the superior efficacy of ibrutinib over conventional chemotherapy in treating MCL, CLL, Waldenstrom macroglobulinemia (WM), small lymphocytic lymphoma (SLL), and marginal zone lymphoma (MZL) [119, 120]. However, the non-specific effects of ibrutinib on other TEC family kinases and EGFR have resulted in adverse events such as joint pain and atrial fibrillation [121, 122]. Next-generation BTK inhibitors, including zanubrutinib and acalabrutinib, have been approved for relapsed/refractory MCL and exhibit enhanced selectivity and fewer off-target effects than ibrutinib [123, 124]. These agents are currently being evaluated in trials for NHL and other malignancies [125]. Emerging third-generation BTK inhibitors, such as tirabrutinib and spebrutinib, are under investigation for their efficacy and safety in various B-cell malignancies, with some demonstrating potent activity against BTK Cys-481 mutants associated with resistance to earlier-generation inhibitors [126, 127]. To address resistance, researchers are developing non-covalent BTK inhibitors (e.g., vecabrutinib and LOXO-305) and targeting upstream signaling pathways such as LYN and SYK, either independently or in combination with BTK inhibitors [125]. These approaches aim to enhance therapeutic efficacy for patients with B-cell malignancies that are refractory to existing treatment modalities.

### Janus kinase (JAK) inhibitors

The Janus kinase (JAK) family comprises four homologous non-receptor tyrosine kinases: JAK1, JAK2, JAK3, and TYK2 [128]. JAK1, JAK2, and TYK2 exhibit ubiquitous distribution, whereas JAK3 is predominantly localized in bone marrow and lymphatic tissues [129]. These enzymes play a crucial role in transducing external signals to the nucleus, where they activate and phosphorylate other signal transducers and transcriptional activators. This signaling cascade is initiated when inflammatory cytokines, including interleukins and interferons, interact with cytokine receptors, resulting in STAT protein phosphorylation and subsequent translocation to the nucleus, where they regulate target gene expression. The selective activation of specific JAKs and STATs by cytokine receptor intracellular domains elucidates the capacity of the pathway to modulate responses to over 50 cytokines and growth factors [129].

The involvement of the JAK/STAT signaling pathway in cytokine signal transduction renders it a promising target for the treatment of autoimmune disorders, such as rheumatoid arthritis and systemic lupus erythematosus. Furthermore, aberrant STAT protein activity, particularly that of STAT3, STAT5, and STAT6, is associated with hematological malignancies and influences cell proliferation, survival, invasion, and inflammation [128]. As upstream regulators of STAT signaling, JAKs are considered potential therapeutic targets in cancer therapy, especially for hematological malignancies.

Currently, four JAK inhibitors have received clinical approval. Ruxolitinib, the first to obtain approval in 2011, primarily targets JAK1, JAK2, and to a lesser extent TYK2, and is utilized in the treatment of conditions such as myelofibrosis, polycythemia vera, and bone marrow malignancies [130, 131]. Fedratinib, another JAK2 inhibitor, has demonstrated efficacy in treating myelofibrosis and has shown promise in pre-clinical NSCLC models, where it can overcome erlotinib resistance and suppress PD-L1, a significant factor in checkpoint immunotherapy [132]. Tofacitinib is employed in the treatment of autoimmune diseases, while baricitinib is utilized for other autoimmune conditions.

Several novel Janus kinase (JAK) inhibitors are currently undergoing clinical trials for cancer therapies. WP1066, a compound that inhibits both the JAK2 and STAT3 pathways, has been used in preclinical testing for melanoma and glioblastoma [133]. Gandotinib, a selective JAK1/JAK2 inhibitor, has demonstrated efficacy in managing Myeloproliferative neoplasm diseases, as reported by Muller and Vousden in 2013. Staurosporine selectively inhibits KIT, JAK2, Bclxl, and XIAP in both experimental and clinical trials. These researchers identified that lestaurtinib (CEP701), which targets JAK2, FLT3, and TrkA, may be beneficial for various cancer types including acute myeloid leukemia (AML) and Hodgkin lymphoma. INCB039110, a selective JAK1 inhibitor, has been shown to alleviate myelofibrosis symptoms. Pacritinib, a dual JAK2/FLT3 inhibitor, is effective in treating myelofibrosis and AML, which are rapidly progressing bone marrow cancers.

JAK inhibitors have demonstrated effectiveness in the treatment of autoimmune disorders, myelofibrosis, and polycythemia vera. Future studies should explore the potential of combining JAK inhibitors with PD-L1 monoclonal antibodies and STAT inhibitors for cancer therapy [133]. Nevertheless, patients receiving JAK inhibitors require careful monitoring and management owing to possible adverse effects, including infections, neutropenia, venous thromboembolism (VTE), and an increased risk of malignancies [134].

### Oligonucleotides

**Fig. 3:**
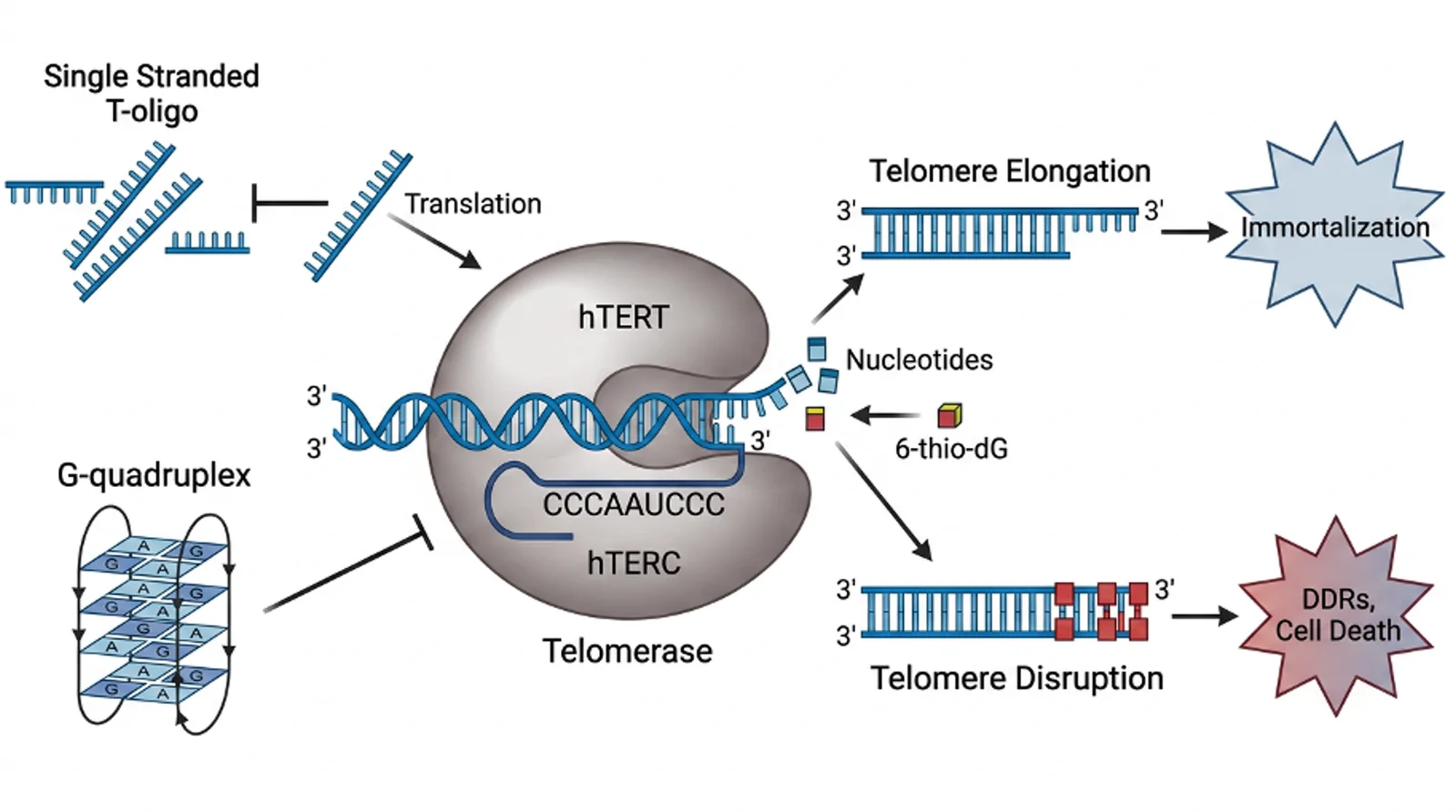
Mechanisms of telomerase inhibition by oligonucleotide-based compounds

Oligonucleotides, short sequences of DNA or RNA capable of binding to complementary sequences - have found extensive applications in medicine and biomedical research. These molecules are instrumental in various processes, including nucleic acid probes, essential laboratory methods such as PCR and DNA sequencing, and the modulation of protein expression and function [135, 136]. Their versatility has rendered them crucial in cancer biology investigations, targeting specific nucleotide sequences implicated in cancer etiology and therapy [137–139]. Certain oligonucleotide compounds, particularly antisense oligodeoxynucleotides, have been utilized to regulate abnormal protein concentrations and enzymatic activities associated with various types of cancers [137, 140, 141]. A notable example is 6-Thio-20-Deoxyguanosine (6-Thio-dG), which functions as a nucleoside analog and telomerase substrate, demonstrating potent anti-cancer effects via DNA incorporation (Fig. 3) [137, 140].

This figure illustrates how T-oligo, 6-thio-dG, and G-quadruplex stabilizing agents (e.g., telomestatin) impede telomere extension. T-oligo induces DNA damage signaling and suppresses hTERT expression via JNK pathway activation, while 6-thio-dG incorporates into telomeric DNA, causing telomere dysfunction-induced foci (TIFs). G-quadruplex stabilizers like telomestatin bind to guanine nucleotides at telomere ends, preventing telomerase access.

Significant advancements have been made in utilizing oligonucleotides to target molecular mechanisms that enhance telomerase activity, with a particular emphasis on the telomerase catalytic subunit (hTERT), the essential catalytic component for telomere replication and cellular proliferation potential [142–144]. Oligonucleotides can be engineered to interfere with hTERT at multiple stages of its synthesis and operation, including by targeting its promoter, mRNA, or the protein itself [137, 145]. Antisense oligodeoxynucleotides (AS-ODNs) have demonstrated efficacy in preclinical and clinical studies across various cancer types by targeting the hTERT initiator sequence, resulting in decreased hTERT activity and enhanced growth inhibition in cancers such as human hepatoma, prostate adenocarcinoma, bladder cancer, and hepatic lymphoma [66, 146–148]. Notably, AS-ODN-based inhibition of hTERT, but not hTR (telomerase RNA component), has been found effective in inducing apoptosis and suppressing cell growth in human prostate cancer cells [146, 147].

AS-ODNs have exhibited efficacy in targeting hTERT at various regulatory stages of its cellular synthesis in preclinical research, indicating their potential for broad applications in addressing cells with abnormal hTERT activity [149]. Synergistic effects between AS-ODNs and other cancer treatments have been investigated in multiple preclinical and clinical studies, revealing enhanced inhibition of aberrant cancer cell proliferation [150–152]. Imetelstat (GRN163L), a first-in-class 13-mer oligonucleotide N3′→P5′ thio-phosphoramidate, is a potent and specific competitive inhibitor of telomerase [153, 154]. This compound features a palmitoyl group attached to the 5′ terminus through an aminoglycerol linker, which enhances cellular entry and its anti-telomerase activity. Imetelstat binds with high affinity to the template region of the human telomerase RNA component (hTR), specifically targeting the 5′-CUAACCCUAAC-3′ template sequence, acting as a direct competitive inhibitor of the enzyme’s catalytic activity [155]. The N3′→P5′ thio-phosphoramidate chemistry confers enhanced nuclease resistance and improved pharmacokinetic properties compared to conventional phosphorothioate oligonucleotides. The palmitoyl lipid modification at the 5′ end further improves cellular uptake and biodistribution, particularly to hematopoietic tissues. This binding prevents telomere elongation, promotes progressive telomere shortening, and induces apoptotic cell death in malignant cells. Furthermore, Imetelstat exhibited anti-cancer progression properties in mouse models of lung cancer [156, 157].

Recent clinical success has been achieved with imetelstat, which received FDA approval in June 2024 for adult patients with lower-risk myelodysplastic syndromes (LR-MDS) with transfusion-dependent anemia. In the pivotal phase 3 IMerge trial, significantly more patients treated with imetelstat achieved red blood cell transfusion independence for ≥ 8 weeks and ≥ 24 weeks compared to placebo. The recommended dosing regimen is 7.1 mg/kg administered via 2-hour intravenous infusion every 4 weeks. The safety profile is manageable, primarily characterized by short-lived and manageable neutropenia and thrombocytopenia. Notably, imetelstat has shown activity across a broad spectrum of molecular mutations, including high-risk alterations, with responses observed regardless of ring sideroblast morphology [155]. Despite promising preclinical results, imetelstat clinical development was challenged by thrombocytopenia and myelosuppression; however, subsequent clinical evidence ultimately supported FDA approval for lower-risk myelodysplastic syndromes in 2024. Diverse outcomes have been reported in clinical trials, with no significant increase in long-term survival or reduction in tumor size, despite substantial telomerase inhibition [158, 159]. Combination therapies using metelstat with 3-aminobenzamide (3AB) and trastuzumab have shown promising results in enhancing cancer cell sensitization to treatment [160, 161]. In addition to AS-ODNs, researchers have shown interest in T-oligos and 6-Thio-dG as potential inhibitors given their capacity to modulate hTERT and their possible use in cancer therapy. T-oligos, which simulate telomeric overhang sequences, trigger DNA damage responses that result in cancer cell death, without harming normal cells [162]. Specific T-oligos, such as T11, have demonstrated anti-cancer properties in various malignancies, including melanoma, by inhibiting hTERT mRNA expression and enhancing the effects of existing chemotherapeutics [163, 164]. 6-Thio-dG, a nucleoside analog, disrupts telomere structure and function, leading to cellular senescence and apoptosis, particularly in cells with high hTERT activity (Fig. 3) [150].

### Quadruplex

The human genome is primarily composed of double-stranded DNA, known as the double helix, but emerging research has uncovered unconventional DNA structures that deviate from traditional Watson-Crick base pairing [165, 166]. Among these, G-quadruplexes (G4s) stand out as complex secondary structures formed by guanine-rich nucleic acid sequences. Stabilized by monovalent ions like Na + and K+, G4s consist of multiple layers of Hoogsteen-bonded guanine tetrads [167, 168]. These structures exhibit greater thermodynamic stability than B-form DNA and RNA hairpins, with slower unfolding rates, enabling them to influence gene transcription, protein translation, and genomic stability [169, 170]. Their regulatory roles underscore the importance of understanding and controlling G4 formation. G4 structures are particularly significant in telomere function and flexibility, offering a unique therapeutic avenue distinct from traditional cancer treatments that rely on external nucleotide structures like miRNAs and oligonucleotides [162, 171]. Genomic sequencing has revealed an abundance of potential G4-forming sequences in DNA, with retroelement G4s frequently found in replication origins [172, 173]. Approximately 90% of replication origins in the human genome contain G4 features, suggesting their regulatory role in DNA replication [174, 175]. Additionally, G4 sequences are associated with chromosomal terminal regions, where they contribute to telomere capping and may regulate telomerase activity [168, 176]. While initially thought to inhibit telomerase, recent studies indicate that telomeric G4 structures can also modulate access to telomerase, influencing telomere elongation [176, 177]. Enzymes like BLM, RTEL1, and WRN facilitate G4 unwinding, ensuring telomere replication and genome stability [178, 179]. Disruptions in these processes can lead to replication stalling, highlighting the potential of G4-stabilizing compounds in cancer therapy [180].

Several compounds, including BRACO-19, RHPS4, and telomestatin, have been explored for their ability to stabilize G4 structures [96, 97]. Telomestatin, for instance, binds to guanine nucleotides at telomere ends, stabilizing G4s and inhibiting telomerase RNA (hTR) and single-stranded telomere overhang binding. Preclinical studies have demonstrated its telomerase-inhibiting effects in breast, cervical, and neuroblastoma cancer cell lines, though its poor water solubility remains a challenge. BRACO-19 and RHPS4 also disrupt G4 structures but lack specificity for tumor cells. Repetitive telomeric G4 sequences make them attractive targets for therapeutic molecules like T-oligo, which exhibits anticancer effects through pathways such as JNK activation and shelterin complex modulation. Recent research has also focused on targeting G4 structures in hTERT promoter mutations, which are prevalent in melanoma and other tumors. Compounds like GTC365 aim to restore wild-type G4 folding in mutated hTERT promoters, reducing telomerase activity and inducing cancer cell death [109].

The study of G4 structures has evolved from structural analyses to exploring their roles in DNA and RNA processes and cancer therapy. Future research should investigate the concentration and functional implications of G4 structures near cancer-associated genes and their mechanisms of transcriptional regulation. For instance, lung cancer, particularly non–small cell lung cancer (NSCLC), remains a leading cause of cancer mortality worldwide. KRAS mutations play a significant role in NSCLC development, immune escape, and chemotherapy resistance. While two KRAS inhibitors have been approved for NSCLC treatment, their efficacy is limited to KRAS G12C mutants, and drug resistance remains a challenge. This study identified a novel tribenzophenazine analog (MBD) as a ligand targeting RNA G-quadruplexes in the 5′-UTR of KRAS mRNA. MBD demonstrated antitumor efficacy in vitro and in vivo, suggesting its potential as a therapeutic agent for KRAS-driven NSCLC.

Similarly, well-differentiated/dedifferentiated liposarcomas (WD/DDLPSs), which account for 60% of liposarcomas, have limited treatment options and a poor prognosis. These tumors are characterized by aberrant expression of MDM2, which forms G4s in its promoter. The naphthalene diimide derivative QN-302 has shown promise in targeting MDM2 G4s, impairing WD/DDLPS cell growth and stabilizing G4s at the P2 promoter. This stabilization prevents polymerase progression, inhibiting full-length MDM2 transcript formation and reactivating p53 through the p53-MDM2 feedback loop, leading to apoptosis. In patient-derived xenograft models, QN-302 reduced tumor volume and was well tolerated, offering a novel therapeutic strategy for tumors with wild-type TP53, such as WD/DDLPSs [181].

Recent advances in G-quadruplex targeting include the identification of novel natural product ligands. Levo-tetrahydropalmatine (l-THP), an isoquinoline alkaloid, and its two demethylated metabolites (M1 and M2) have been shown to bind to the hTERT promoter G-quadruplex with moderate affinity, suppressing hTERT mRNA expression (0.43 ± 0.02 for l-THP, 0.19 ± 0.01 for M1, 0.33 ± 0.03 for M2) and exhibiting potent antitumor activity in A549 cells and lung cancer xenograft models [182]. Fluorescence mutation assays revealed preferential interaction at the junction between the first and second G-tracts, particularly recognizing cytosine at position 21. Computational studies have also provided mechanistic insights into small molecules like Triethylenetetramine (TETA) binding to G-quadruplexes, demonstrating that conformational flexibility is crucial for specific and stable interactions within the G-quadruplex cavity, leading to enhanced stabilization and functional inhibition of telomerase [183].

## Advancing cancer immunotherapy: the telomerase catalytic subunit (hTERT) as a target antigen

**Fig. 4:**
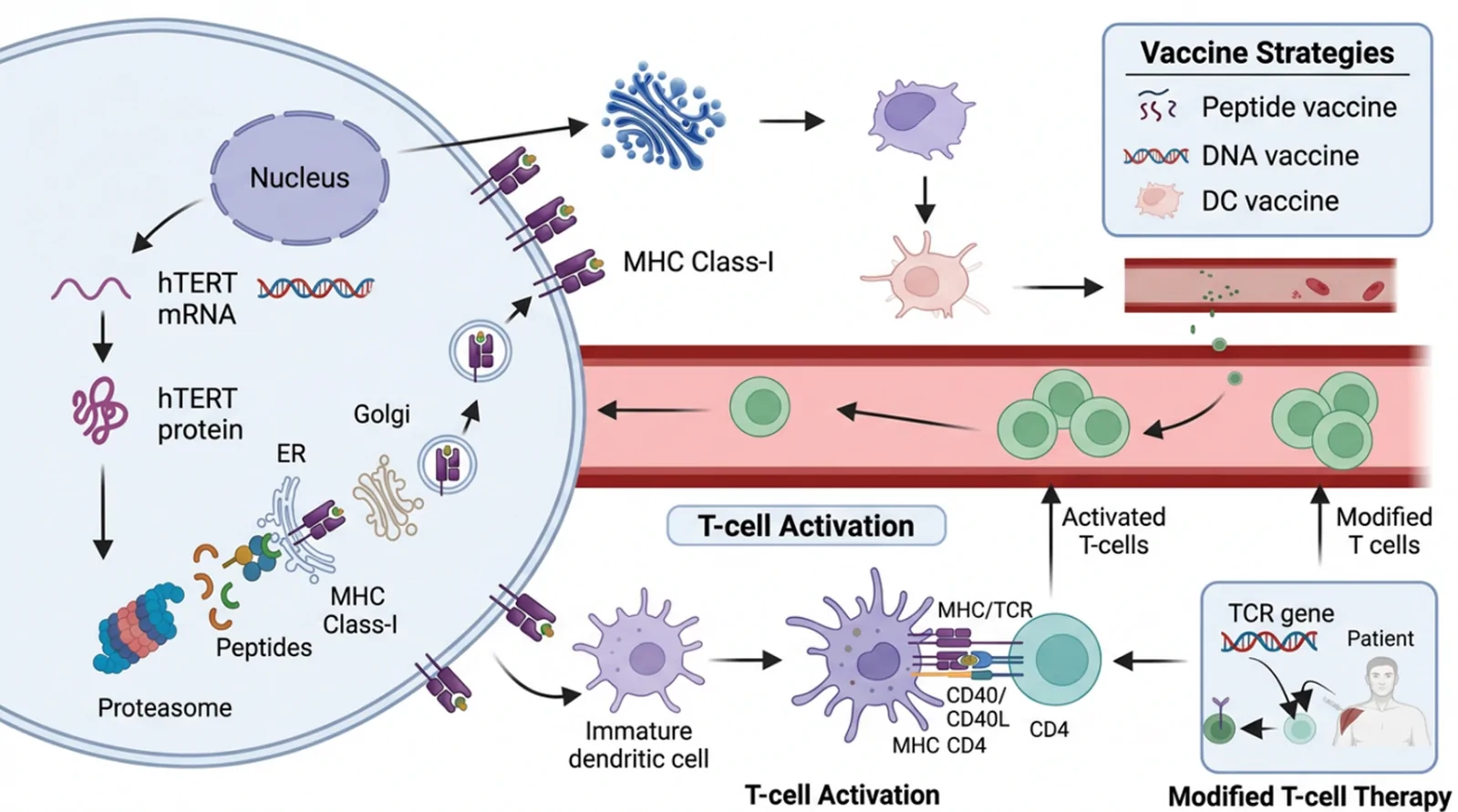
The immune response cycle specific to the telomerase catalytic subunit (hTERT) and telomerase-targeted immunotherapy in cancer management

Immunotherapy targeting tumor-specific immune responses commences with the detection of tumor-associated antigens (TAAs), including cancer/testis antigen and carcinoembryonic antigen (CEA) [184, 185]. Upon cellular death, cancer cells release these TAAs, which are subsequently captured by dendritic cells. These antigens undergo processing into peptides capable of binding to major histocompatibility complex (MHC) molecules on the dendritic cell surface. Peptides of greater length are present in MHC class II molecules, which subsequently activate CD4 + helper T cells. Conversely, shorter peptides bind to MHC class I molecules, eliciting CD8 + cytotoxic T-lymphocytes (CTLs), which play a crucial role in cancer cell elimination (Fig. 4) [186].

This figure illustrates the process by which hTERT is released from cancer cells, captured by dendritic cells, and presented via MHC molecules to activate CD8 + cytotoxic T lymphocytes and CD4 + helper T cells. These activated T cells then target and eliminate hTERT-expressing cancer cells.

Novel tumor-associated antigens (TAAs) are frequently identified using methodologies such as serological analysis of recombinant cDNA expression libraries (SEREX) and cDNA microarrays. A notable example is human telomerase reverse transcriptase (hTERT), which exhibits elevated expression in various neoplasms and certain normal cells, including those in the testes, rendering it a promising target for immunotherapy [6, 187, 188]. As a TAA present throughout the body, hTERT-derived peptides can interact with major histocompatibility complex (MHC) molecules, eliciting responses from both cytotoxic T lymphocytes (CTLs) and helper T cells. Numerous peptides originating from hTERT have demonstrated potential as cancer-associated antigens that may be utilized in immunotherapeutic strategies.

## Strategic peptide vaccination against the telomerase catalytic subunit (hTERT)

Studies have been conducted on hTERT-derived peptides to evaluate their safety, immunogenicity, and efficacy as antitumor vaccines for various cancer types. These vaccines exhibit high tumor specificity and contain both MHC I and MHC II epitopes within their amino acid sequences. Currently, more than 30 hTERT peptides function as epitope mimics. The majority of these peptides are presented on MHC class I molecules, promoting enhancement of CTL response, while others are displayed on MHC class II molecules, stimulating Th cell response [189].

### GV1001-vaccine

GV1001, a peptide vaccine derived from the hTERT active site (611–626, EARPALLTSRLRFIPK), is MHC class II-restricted and necessitates an adjuvant, such as GM-CSF or TLR-7. It elicits significant CD4 + and CD8 + T-cell proliferation and enhances cytotoxic T lymphocyte (CTL)-mediated target cell destruction. Moreover, GV1001 exhibited cellular effects, including cell penetration and cytoplasmic localization. It inhibits HSPs, such as HSP90 and HSP70, as well as proteins, including HIF-1α and VEGF, in hypoxic tumor microenvironments, which elucidates its notable anti-tumor activity [190, 191].

In renal cell carcinoma, GV1001 inhibits cancer cell growth, induces apoptosis, and suppresses angiogenesis [192]. It also exhibits anti-inflammatory [193–195] and antiviral properties [196, 197]. Furthermore, GV1001 has neuroprotective effects against β-amyloid toxicity in the central nervous system [198]. Among the hTERT-targeting vaccines, GV1001 has undergone various clinical trials for different cancers, including pancreatic cancer, NSCLC, and melanoma. Initial phases revealed that 50–80% of patients with advanced pancreatic and lung cancer can activate specific T-cells, with the treatment demonstrating safety [199]. Notably, GV1001 can be administered without HLA type-based patient selection [200].

Combining GV1001 with other peptide vaccines, such as the HLA-A 2 restricted CTL epitope HR2822 (hTERT 540–548), yielded promising results. This combination induced immune responses in 86% of patients and has been developed for NSCLC patients, with one patient exhibiting a complete response [201]. At the molecular level, GV1001 targets antigen-presenting cells (APCs) in tumors and lymph nodes where cancer cells are detected but not in the bone marrow [201]. Some combinations, such as GV1001 with gemcitabine for pancreatic ductal carcinoma, effectively promote tumor cell death [202, 203]. However, a phase III trial investigating the combination of GV1001 and chemotherapy in metastatic pancreatic cancer demonstrated no improvement in overall patient survival [203]. Recent clinical trials have further investigated the efficacy of GV1001 in combination with gemcitabine/capecitabine in patients with advanced pancreatic ductal adenocarcinoma, focusing on biomarkers such as serum eotaxin levels to predict response [204].

### GX301-vaccine

GX301 is a vaccine composed of four hTERT peptides: hTERT540-548, hTERT611-626, hTERT672-686, and hTERT766-780. This vaccine exhibits the capacity to bind to MHC class I and II, as well as adjuvants such as montanide ISA-51 and Imiquimod. A phase I clinical trial involving patients with stage IV prostate and kidney cancer revealed that all participants exhibited an immune response to at least one peptide. This observation suggests that vaccines containing multiple peptides are more efficacious than those containing a single peptide, as they elicit a more robust immune response in a larger number of individuals who respond to treatment [205].

### UV1-vaccine

Recent investigations have yielded findings on UV1, a second-generation telomerase peptide vaccine. This vaccine comprises three peptides derived from hTERT, which are frequently observed in cancer patients with long-term survival: hTERT652-665 and hTERT660-689 (ALFSVLNYERARRPGLLGASVLGLDDIHRA) and hTERT691-705 (RTFVLRVRAQDPPPE). A phase I/II study evaluated the vaccine in combination with GM-CSF as an immunomodulator for a six-month period in patients with metastatic hormone-naïve prostate cancer. The results demonstrated a reduction in PSA levels in 64% of the patients and an antibody response in 85. Further MRI analysis of the same patient cohort revealed that prostate tumors disappeared in 45% of patients following immunization. Most reported adverse effects were classified as grade I [206]. Recent studies combining UV1 with immune checkpoint inhibitors such as ipilimumab and nivolumab in patients with malignant mesothelioma have demonstrated rapid immune responses and clinical benefit [207].

### Vx-001-vaccine

Vx-001 is a vaccine comprising two peptides derived from the hTERT protein. One is an optimized hepta-peptide form of the intermediate-affinity cryptic hTERT peptide (YLFFYRKSV) and the other is RLFFYRKSV. The latter, featuring a tyrosine residue as the first amino acid, demonstrates enhanced affinity for MHC class I molecules. Phase I/II clinical trials have demonstrated Vx-001’s antitumor effects in various cancers including melanoma, breast carcinoma, bile duct carcinoma, and NSCLC. These studies also revealed that the vaccine elicits a robust hTERT-specific immune response, yielding the most promising clinical outcomes. Moreover, Vx-001 exhibited a favorable safety profile, with patients experiencing only mild adverse effects [208, 209].

### Harnessing telomerase catalytic subunit (hTERT)-targeting dendritic cells for innovative immunotherapy

Among all antigen-presenting cells, dendritic cells (DCs) are the most efficacious because of their role in both adaptive and innate immunity. Over the past decade, DCs have been used to elicit potent antitumor immune responses in cancer immunotherapy. The United States Food and Drug Administration approved a DC vaccine called sipuleucel-T for the treatment of metastatic prostate cancers. Sipuleucel-T, an autologous cellular therapy derived from culturing DCs with a tumor antigen (PAP fusion protein), has been demonstrated in a phase III trial to extend survival by approximately four months [209]. Furthermore, DCs have been used in research on hTERT-targeted immunotherapy.

### GRNVAC1-vaccine

In Washington, DC, a novel cancer vaccine designated as GRNVAC1 has been developed. This vaccine utilizes mRNA encoding hTERT and LAMP1, which are introduced into autologous mature dendritic cells (DCs). LAMP1 facilitates the transport of hTERT to lysosomes, where it undergoes proteolysis to form smaller peptides. This process activates T cells against various epitopes of the hTERT peptide, thereby eliciting a robust immune response. Studies in animal models and clinical trials have demonstrated that GRNVAC1 is both safe and efficacious with minimal adverse effects. Notably, patients with metastatic prostate cancer who received DCs transfected with the LAMP1-hTERT mRNA chimera did not exhibit autoimmune reactions, even after multiple administrations. The vaccine was found to activate both CD8 + and CD4 + T cells [208]. Current research suggests that GRNVAC1 may have potential applications in the treatment of Acute Myeloid Leukemia [208]. GRNVAC2, which is structurally analogous to GRNVAC1, is derived from human embryonic stem cells, instead of leukapheresis-derived DCs. This vaccine demonstrates potential, particularly in terms of delivery systems [199].

Researchers have investigated various approaches to DC-based vaccines that target hTERT. One example involves an IDO-shrunken DC vaccine expressing survivin and hTERT mRNA, which generated tumor-specific T cells against these proteins in patients with metastatic melanoma treated with ipilimumab. Additionally, T-cell responses to MART-1 and NY-ESO-1 were observed, correlating with reduced organ metastases and improved patient performance status [210]. Another strategy employed a recombinant adenovirus carrying hTERT cDNA to transfect DCs, resulting in the generation of hTERT-specific CTLs in cultures, indicating enhanced CTL reactivity [211].

### TAPCells-based vaccine

A sophisticated approach utilizing dendritic cells involves the development of TAPCells, which are therapeutic dendritic-like cells that function as tumor antigen-presenting cells. This TAPCell-based vaccine has been evaluated in multiple phase I and phase I/II clinical trials involving over 120 patients with stage III and IV melanoma and 20 individuals with castration-resistant prostate cancer. These studies demonstrated the significant clinical benefits of the TAPCell vaccine. Melanoma patients experienced an increased survival time, while prostate cancer patients exhibited a doubling in the time required for their PSA levels to double. Notably, the vaccine induced high-quality T cells in patients with prostate cancer. Furthermore, more than 60% of the patients exhibited DTH reactions against the lysates used in the vaccine, indicating the formation of antitumor immune memory, which is a crucial factor for long-term therapeutic efficacy. Research also revealed that vaccination led to an increase in certain immune cells, such as Th1 and Th17 cells, which are associated with antitumor responses. The study concluded that CCH, when used as an adjuvant, is safe and further enhances the immune response [208].

### Unconventional DC-based strategies

A 2014 phase I study by Mehrotra et al. investigated the use of three A2-restricted peptides (hTERT572Y, Cap1-6D, and survivin) administered via dendritic cells (DCs) for treating pancreatic cancer. This DC vaccine aimed to elicit specific T-cell responses. The results indicated that 50% of the patients exhibited stable disease (SD), with a median overall survival of 7 months. The vaccine demonstrated favorable tolerability, with the majority of patients experiencing mild adverse effects such as fatigue and influenza-like symptoms [209].

No severe adverse events were reported regarding the safety profile of hTERT-targeted immunotherapy. However, it is imperative to consider its potential impact on the host’s immune system. Hematopoietic progenitor cells, B cells, and T cells express elevated levels of telomerase, rendering them potential targets for hTERT-based therapeutic interventions.

## DNA vaccine

### phTERT-vaccine

The utilization of recombinant DNA methodologies presents a promising strategy for enhancing the display of telomerase catalytic subunit (hTERT) epitopes on T cells, potentially augmenting the efficacy of immunotherapy. This approach involves the introduction of plasmids containing hTERT genomes into antigen-presenting cells using techniques such as gene guns or electroporation. DNA-based vaccines frequently demonstrate greater cost-effectiveness than their peptide-based counterparts.

A notable example is the phTERT vaccine, which is a full-length DNA vaccine encoding hTERT that has been optimized for expression. Preclinical trials in murine models and nonhuman primates have demonstrated that phTERT, when administered via electroporation, can elicit robust and diverse CD8 + T cell responses specific to hTERT. These T cells exhibit cytotoxic activity indicators such as CD107a expression and produce IFN-γ and TNF-α. Moreover, vaccinated primates exhibited significant IFN-γ responses and specific perforin release, suggesting potent in vivo cytotoxicity against hTERT-expressing cells, effectively overcoming immune tolerance [210]. Furthermore, studies utilizing HPV-related tumor models have demonstrated the preventive and therapeutic potential of phTERT. The vaccine was observed to decelerate tumor growth and enhance survival rates, underscoring its efficacy in a clinically relevant context [210]. Recent advances in DNA vaccines include INO-5401, a plasmid DNA-based immunotherapy encoding hTERT, prostate-specific membrane antigen (PSMA), and Wilms Tumor-1 (WT1). A phase Ib study (NCT04367675) demonstrated that INO-5401, with or without the IL-12-encoding plasmid INO-9012, administered via intramuscular injection followed by electroporation, is feasible and safe in individuals with BRCA1/2 mutations, including healthy individuals with no prior cancer. The most common adverse events were Grade 1/2 injection site reactions, with no treatment-related Grade 3 events reported [212]. This study supports the potential of DNA-based hTERT-targeting vaccines for cancer interception in high-risk populations.

## Innovative immunotherapy: cell-based strategies and advances

Researchers have used human umbilical vein endothelial cells (HUVECs) in cell-based immunotherapy approaches by genetically modifying them with hTERT genes through lentiviral transduction. These modified cells exhibit enhanced telomerase activity and express essential markers including CD31, VEGF receptor-2 (VEGFR-2), and integrin α5. In animal studies, these engineered HUVECs were irradiated to inhibit their proliferation and used as vaccines. When administered subcutaneously in mouse models of lung and colorectal cancer, this vaccine elicited robust antibodies and cellular immune responses. These findings suggest promising tumor-suppressive and preventive effects [213].

An alternative innovative approach utilizes adenovirus as a vector for a combination vaccine that targets both hTERT and VEGFR-2. This vaccine, developed using a mannan-modified adenovirus, has demonstrated efficacy in inducing potent anti-tumor immune responses. Stimulating cytotoxic T lymphocytes (CTLs) that specifically target hTERT and VEGFR-2 effectively inhibits angiogenesis within tumors. This dual-targeting strategy demonstrates the potential for cancer treatment by simultaneously targeting tumor cells and their vascular supply [214].

## T-cell transformation therapy

Genetically modified T cell therapy is a methodology for delivering T cells with the capacity to target diverse cancer types. This approach employs T cells genetically engineered to express T-cell receptors (TCRs) that recognize specific tumor antigens and their epitopes. Currently, two methods exist for generating gene-modified T cells: the isolation of tumor antigen-specific TCRs from T cells that have infiltrated tumors or their clones [215, 216], and the use of chimeric antigen receptors [217]. The extracellular component of CAR comprises a single-chain antigen recognition receptor, consisting of the variable regions of the heavy and light chains of a monoclonal antibody that are specific to a tumor surface antigen. The intracellular portion of CAR is constructed by linking co-stimulatory molecules to the intracellular segment of TCR.

TCR-engineered T (TCR-T) cells are generated by introducing the genome of TCRs that recognize the tumor peptide antigen/MHC complex into T cells. Consequently, TCR-T therapy is dependent on the presence of target antigen epitopes and MHC molecules in tumor cells. Previous investigations have demonstrated that numerous carcinoma cells exhibit epitopes derived from human telomerase reverse transcriptase (hTERT). Therefore, TCR-T cell-based immunotherapy targeting these epitopes may be beneficial in patients with hTERT-expressing cancers. Other researchers have identified TCRs for the telomerase catalytic subunit and proposed their potential application in immunotherapy [218, 219].

## Telomeres and telomerase: innovative applications in clinical oncology

Although significant progress has been made in elucidating the role of telomerase in cancer, translating this knowledge into effective treatments remains a substantial challenge. Cancer cells typically exhibit shorter telomeres than normal tissues, although the relationship between telomere length and cancer progression is highly variable across cancer types [220, 221]. Telomere length in tumors has been identified as a predictor of patient survival and treatment response [216, 222]. Additionally, specific telomere maintenance mechanisms (TMMs) and telomerase gene mutations provide crucial insights into potential therapeutic targets [142, 223].

Current treatment approaches targeting telomerase include the use of enzyme inhibitors and cytotoxic substrates that focus on telomerase activity. These methods have demonstrated potential in preclinical studies, and have progressed to some extent in clinical trials [157, 224, 225]. However, tumors can activate alternative TMMs, such as alternative lengthening of telomeres (ALT), in response to treatment, potentially diminishing the therapeutic efficacy [32, 34].

Immunotherapy is a novel approach for targeting telomerase by treating it as a neoantigen. These strategies include peptide vaccines and dendritic cell-based vaccines designed to elicit immune responses against telomerase-expressing cancer cells [205, 218, 219]. By leveraging the capacity of the immune system to identify and target cancer cells, these approaches aim to overcome the limitations associated with conventional therapies.

Despite these advancements, significant challenges remain in fully elucidating the complex roles of telomerase in cancer biology and developing therapies that can effectively address tumor heterogeneity and treatment resistance [226, 227].

## Conclusion

Targeting telomerase has emerged as a highly promising strategy in anticancer therapy, as evidenced by the expanding arsenal of therapeutic modalities including small-molecule inhibitors, oligonucleotide-based therapies, and immunotherapeutic vaccines. This review has elucidated the efficacy of various approaches, such as tyrosine kinase inhibitors, antisense oligonucleotides like imetelstat (which received FDA approval in 2024 for lower-risk MDS), and peptide/DNA vaccines, in reducing telomerase activity and impeding tumor growth. These agents offer a targeted approach that may minimize deleterious effects on healthy cells, presenting a potentially advantageous alternative to conventional cytotoxic chemotherapy. Nevertheless, the clinical translation of telomerase-targeted therapies faces persistent challenges, including the development of resistance mechanisms, the emergence of alternative telomere maintenance pathways such as ALT, and the potential for off-target effects.

The implications for future research are substantial. Further studies are necessary to elucidate the complex interactions among various inhibitors, G-quadruplex ligands, cell-based vaccines, telomerase components, and telomere structures. Recent advances in targeting the hTERT promoter G-quadruplex with small molecules like l-THP and its derivatives, as well as the development of novel DNA vaccines (e.g., INO-5401) for cancer interception in high-risk populations, highlight the rapid evolution of this field. Understanding these relationships is essential to enhance telomerase inhibition strategies and overcome challenges such as drug resistance and the emergence of alternative telomere maintenance mechanisms.

In clinical research, telomerase inhibitors represent a novel approach for cancer management. However, several critical questions remain unanswered, including determining the optimal treatment duration, addressing the adverse effects of extreme telomere shortening on normal tissues, mitigating the long-term consequences of telomerase inactivation on stem cell function, and managing the myelosuppressive effects observed with agents like imetelstat. These issues require further investigation through clinical trials and extended follow-up studies to confirm the safety and efficacy of these therapies. Additionally, the identification of reliable predictive biomarkers to select patients most likely to benefit from telomerase-targeted therapies remains a priority for personalized medicine approaches.

In summary, targeting telomerase remains a highly promising approach for anticancer treatment. Advancements in small-molecule inhibitors, oligonucleotide-based therapies, and immunotherapeutic strategies continue to highlight the potential of telomerase targeting. Although significant obstacles persist, the recent FDA approval of imetelstat for lower-risk MDS represents a landmark achievement that validates the clinical feasibility of telomerase inhibition. Ongoing developments in this field may lead to improved cancer management, potentially increasing survival rates and achieving long-term remission for patients.

## Data Availability

The authors confirm that the data presented in the article and additional data can be provided by the corresponding author upon request.

## Abbreviations

3AB: 3-Aminobenzamide
6-Thio-dG: 6-Thio-2′-Deoxyguanosine
ALL: Acute Lymphoblastic Leukemia
ALK: Anaplastic Lymphoma Kinase
ALT: Alternative Lengthening of Telomeres
AML: Acute Myeloid Leukemia
APB: ALT-Associated PML Body
BBB: Blood-Brain Barrier
BCR: B-Cell Receptor
BTK: Bruton's Tyrosine Kinase
CAR: Chimeric Antigen Receptor
CB: Cajal Body
CEA: Carcinoembryonic Antigen
CLL: Chronic Lymphocytic Leukemia
CML: Chronic Myeloid Leukemia
CTL: Cytotoxic T Lymphocyte
DC: Dendritic Cell
DDR: DNA Damage Response
DLBCL: Diffuse Large B-Cell Lymphoma
DTC: Differentiated Thyroid Cancer
EGFR: Epidermal Growth Factor Receptor
FDA: Food and Drug Administration
FGFR: Fibroblast Growth Factor Receptor
FLT3: FMS-Like Tyrosine Kinase 3
G4: G-Quadruplex
GCT: Giant Cell Tumor
GIST: Gastrointestinal Stromal Tumor
HCC: Hepatocellular Carcinoma
HGF: Hepatocyte Growth Factor
hTERT: Human Telomerase Reverse Transcriptase (Telomerase Catalytic Subunit)
hTR: Human Telomerase RNA Component
ITD: Internal Tandem Duplication
JAK: Janus Kinase
MAPK: Mitogen-Activated Protein Kinase
MCL: Mantle Cell Lymphoma
mCRC: Metastatic Colorectal Cancer
MDS: Myelodysplastic Syndrome
MHC: Major Histocompatibility Complex
MPN: Myeloproliferative Neoplasm
MTC: Medullary Thyroid Cancer
MZL: Marginal Zone Lymphoma
NHL: Non-Hodgkin's Lymphoma
NMPA: National Medical Products Administration
NSCLC: Non-Small Cell Lung Cancer
NTRK: Neurotrophic Tyrosine Receptor Kinase
OS: Overall Survival
PCR: Polymerase Chain Reaction
PDGFR: Platelet-Derived Growth Factor Receptor
PFS: Progression-Free Survival
PI3K: Phosphoinositide 3-Kinase
PSA: Prostate-Specific Antigen
PSMA: Prostate-Specific Membrane Antigen
RCC: Renal Cell Carcinoma
SEREX: Serological Analysis of Recombinant cDNA Expression Libraries
SLL: Small Lymphocytic Lymphoma
STAT: Signal Transducer and Activator of Transcription
TAA: Tumor-Associated Antigen
TAPCell: Therapeutic Antigen-Presenting Cell
TCR: T-Cell Receptor
TEC: Tyrosine Kinase Expressed in Hepatocellular Carcinoma
TERT: Telomerase Reverse Transcriptase (Catalytic Subunit)
TKD: Tyrosine Kinase Domain
TKI: Tyrosine Kinase Inhibitor
TLR: Toll-Like Receptor
TMM: Telomere Maintenance Mechanism
TRK: Tropomyosin Receptor Kinase
VEGFR: Vascular Endothelial Growth Factor Receptor
VTE: Venous Thromboembolism
WD/DDLPS: Well-Differentiated/Dedifferentiated Liposarcoma
WM: Waldenstrom Macroglobulinemia
WT1: Wilms Tumor-1
